# Supplementary material for: Targeted psychological and psychosocial interventions for auditory hallucinations in persons with psychotic disorders: Protocol for a systematic review and meta-analysis
Source: PLoS One. 2024 Jul 3;19(7):e0306324. doi: 10.1371/journal.pone.0306324 (PMC11221679; doi:10.1371/journal.pone.0306324)
Supplement: S1 Table — (DOCX) [file pone.0306324.s002.docx]

**S1 Table**

*Draft of search strategies that will be applied to data bases, examples.*

| **MEDLINE** | | |
| --- | --- | --- |
| *Search Number* | *Search Details* | *Results* |
| #1 | (MH "Hallucinations+") |  |
| #2 | (MH "Hallucinations+") OR (auditory hallucination* OR hallucination* OR verbal auditory hallucination* OR hearing voice*) |  |
| #3 | (MH "Schizophrenia+") OR (MH "Schizophrenia Spectrum and Other Psychotic Disorders+") |  |
| #4 | ( (MH "Schizophrenia+") OR (MH "Schizophrenia Spectrum and Other Psychotic Disorders+") ) OR ( psychosis OR psychoses OR schizophrenia OR schizophrenia spectrum disorder* OR psychotic disorder* OR non-affective psychosis OR schizo* OR schizoaffective disorder* ) |  |
| #5 | (MH "Psychotherapy+") |  |
| #6 | (MM "Psychosocial Intervention") |  |
| #7 | (MH "Cognitive Behavioral Therapy+") |  |
| #8 | (psychological treatment OR psychotherapy OR psychological intervention OR psychosocial intervention OR therapy OR therapies OR treatment OR abreaction OR acceptance and commitment therapy OR acting out OR adlerian OR analytical psychotherapy OR analytical psychotherapies OR anger control OR anger management OR animal therapy OR animal therapies OR art therapy OR art therapies OR assertive training OR assertiveness training OR attention training technique OR autogenic training OR autosuggestion OR aversion therapy OR aversion therapies OR balint group OR befriending OR behavior contracting OR behavior modification OR behavior regulation OR behavior therapy OR behavior therapies OR behaviour contracting OR behaviour modification OR behaviour regulation OR behaviour therapy OR behaviour therapies OR bibliotherapy OR bibliotherapies OR biofeedback OR body psychotherapy OR body psychotherapies OR brief psychotherapy OR brief psychotherapies OR caregiver support OR cbt OR client centre OR client center OR cognitive behavior OR cognitive behaviorial OR cognitive intervention OR cognitive interventions OR cognitive rehabilitation OR cognitive remediation OR cognitive technique OR cognitive techniques OR cognitive therapy OR cognitive therapies OR cognitive treatment OR cognitive treatments OR compassionate mind training OR conjoint therapy OR conjoint therapies OR contingency management OR conversational therapy OR conversational therapies OR conversion therapy OR conversion therapies OR coping skills OR counseling OR counselling OR countertransference OR couples therapy OR couples therapies OR covert sensitization OR covert sensitisation OR crisis intervention OR dance therapy OR dance therapies OR dialectic OR dialectical OR dream analysis OR eclectic OR emotion focused OR emotionally focused OR emotional freedom technique OR encounter group therapy OR encounter group therapies OR existential therapy OR existential therapies OR experiential psychotherapy OR experiential psychotherapies OR exposure therapy OR exposure therapies OR expressive psychotherapy OR expressive psychotherapies OR eye movement desensitization OR eye movement desensitisation OR family intervention OR family interventions OR family therapy OR family therapies OR feminist therapy OR feminist therapies OR free association OR freudian OR geriatric psychotherapy OR geriatric psychotherapies OR gestalt therapy OR gestalt therapies OR griefwork OR group intervention OR group interventions OR group psychotherapy OR group psychotherapies OR group therapy OR group therapies OR guided imagery OR holistic psychotherapy OR holistic psychotherapies OR humanistic psychotherapy OR humanistic psychotherapies OR hypnosis OR hypnotherapy OR hypnotherapies OR hypnotizability OR hypnotisability OR imagery OR implosive therapy OR implosive therapies OR individual psychotherapy OR individual psychotherapies OR insight therapy OR insight therapies OR integrated psychological therapy OR integrative psychotherapy OR integrative psychotherapies OR integrative therapy OR integrative therapies OR interpersonal OR jungian OR kleinian OR logotherapy OR marathon group therapy OR marathon group therapies OR marital therapy OR marital therapies OR meditation OR mental healing OR metacognitive therapy OR metacognitive therapies OR metacognitive training OR milieu therapy OR milieu therapies OR mindfulness OR morita therapy OR morita therapies OR multimodal OR music therapy OR music therapies OR narrative therapy OR narrative therapies OR nondirective therapy OR nondirective therapies OR object relations OR person centred therapy OR person centred therapies OR person centered therapy OR person centered therapies OR personal construct therapy OR personal construct therapies OR persuasion therapy OR persuasion therapies OR pet therapy OR pet therapies OR play therapy OR play therapies OR primal therapy OR primal therapies OR problem solving OR psychoanalyse OR psychoanalysed OR psychoanalysis OR psychoanalytic OR psychodrama OR psychodynamic OR psychoeducate OR psychoeducation OR psychoeducating OR psychologic OR psychological OR psychologically OR psychological therapy OR psychological therapies OR psychosocial treatment OR psychosocial treatments OR psychotherapy OR psychotherapies OR psychotherapeutic counsel OR psychotherapeutic counseling OR psychotherapeutic counselling OR psychotherapeutic processes OR psychotherapeutic training OR psychotherapeutic treatment OR psychotherapeutic treatments OR rational emotive OR reality therapy OR reality therapies OR reciprocal inhibition OR rehabilitation OR rehabilitating OR relationship therapy OR relationship therapies OR relaxation OR reminiscence therapy OR reminiscence therapies OR rogerian OR role play OR role plays OR role playing OR self analysis OR self analysing OR self esteem OR sensitivity training OR sex therapy OR sex therapies OR sleep phase chronotherapy OR sleep phase chronotherapies OR social skills education OR social skills training OR socioenvironmental therapy OR socioenvironmental therapies OR sociotherapy OR solution focused OR stress management OR support group OR support groups OR supportive therapy OR supportive therapies OR systematic desensitization OR systematic desensitisation OR systemic therapy OR systemic therapies OR therapeutic community OR therapeutic communities OR transactional analysis OR transference OR transtheoretical OR validation therapy OR validation therapies) |  |
| #9 | ( (MH "Psychotherapy+") OR (MM "Psychosocial Intervention") OR (MH "Cognitive Behavioral Therapy+") ) OR ( psychological treatment OR psychotherapy OR psychological intervention OR psychosocial intervention OR therapy OR therapies OR treatment OR abreaction OR acceptance and commitment therapy OR acting out OR adlerian OR analytical psychotherapy OR analytical psychotherapies OR anger control OR anger management OR animal therapy OR animal therapies OR art therapy OR art therapies OR assertive training OR assertiveness training OR attention training technique OR autogenic training OR autosuggestion OR aversion therapy OR aversion therapies OR balint group OR befriending OR behavior contracting OR behavior modification OR behavior regulation OR behavior therapy OR behavior therapies OR behaviour contracting OR behaviour modification OR behaviour regulation OR behaviour therapy OR behaviour therapies OR bibliotherapy OR bibliotherapies OR biofeedback OR body psychotherapy OR body psychotherapies OR brief psychotherapy OR brief psychotherapies OR caregiver support OR cbt OR client centre OR client center OR cognitive behavior OR cognitive behaviorial OR cognitive intervention OR cognitive interventions OR cognitive rehabilitation OR cognitive remediation OR cognitive technique OR cognitive techniques OR cognitive therapy OR cognitive therapies OR cognitive treatment OR cognitive treatments OR compassionate mind training OR conjoint therapy OR conjoint therapies OR contingency management OR conversational therapy OR conversational therapies OR conversion therapy OR conversion therapies OR coping skills OR counseling OR counselling OR countertransference OR couples therapy OR couples therapies OR covert sensitization OR covert sensitisation OR crisis intervention OR dance therapy OR dance therapies OR dialectic OR dialectical OR dream analysis OR eclectic OR emotion focused OR emotionally focused OR emotional freedom technique OR encounter group therapy OR encounter group therapies OR existential therapy OR existential therapies OR experiential psychotherapy OR experiential psychotherapies OR exposure therapy OR exposure therapies OR expressive psychotherapy OR expressive psychotherapies OR eye movement desensitization OR eye movement desensitisation OR family intervention OR family interventions OR family therapy OR family therapies OR feminist therapy OR feminist therapies OR free association OR freudian OR geriatric psychotherapy OR geriatric psychotherapies OR gestalt therapy OR gestalt therapies OR griefwork OR group intervention OR group interventions OR group psychotherapy OR group psychotherapies OR group therapy OR group therapies OR guided imagery OR holistic psychotherapy OR holistic psychotherapies OR humanistic psychotherapy OR humanistic psychotherapies OR hypnosis OR hypnotherapy OR hypnotherapies OR hypnotizability OR hypnotisability OR imagery OR implosive therapy OR implosive therapies OR individual psychotherapy OR individual psychotherapies OR insight therapy OR insight therapies OR integrated psychological therapy OR integrative psychotherapy OR integrative psychotherapies OR integrative therapy OR integrative therapies OR interpersonal OR jungian OR kleinian OR logotherapy OR marathon group therapy OR marathon group therapies OR marital therapy OR marital therapies OR meditation OR mental healing OR metacognitive therapy OR metacognitive therapies OR metacognitive training OR milieu therapy OR milieu therapies OR mindfulness OR morita therapy OR morita therapies OR multimodal OR music therapy OR music therapies OR narrative therapy OR narrative therapies OR nondirective therapy OR nondirective therapies OR object relations OR person centred therapy OR person centred therapies OR person centered therapy OR person centered therapies OR personal construct therapy OR personal construct therapies OR persuasion therapy OR persuasion therapies OR pet therapy OR pet therapies OR play therapy OR play therapies OR primal therapy OR primal therapies OR problem solving OR psychoanalyse OR psychoanalysed OR psychoanalysis OR psychoanalytic OR psychodrama OR psychodynamic OR psychoeducate OR psychoeducation OR psychoeducating OR psychologic OR psychological OR psychologically OR psychological therapy OR psychological therapies OR psychosocial treatment OR psychosocial treatments OR psychotherapy OR psychotherapies OR psychotherapeutic counsel OR psychotherapeutic counseling OR psychotherapeutic counselling OR psychotherapeutic processes OR psychotherapeutic training OR psychotherapeutic treatment OR psychotherapeutic treatments OR rational emotive OR reality therapy OR reality therapies OR reciprocal inhibition OR rehabilitation OR rehabilitating OR relationship therapy OR relationship therapies OR relaxation OR reminiscence therapy OR reminiscence therapies OR rogerian OR role play OR role plays OR role playing OR self analysis OR self analysing OR self esteem OR sensitivity training OR sex therapy OR sex therapies OR sleep phase chronotherapy OR sleep phase chronotherapies OR social skills education OR social skills training OR socioenvironmental therapy OR socioenvironmental therapies OR sociotherapy OR solution focused OR stress management OR support group OR support groups OR supportive therapy OR supportive therapies OR systematic desensitization OR systematic desensitisation OR systemic therapy OR systemic therapies OR therapeutic community OR therapeutic communities OR transactional analysis OR transference OR transtheoretical OR validation therapy OR validation therapies ) |  |
| #10 | ( (MH "Hallucinations+") OR (auditory hallucination* OR hallucination* OR verbal auditory hallucination* OR hearing voice*) ) AND ( ( (MH "Schizophrenia+") OR (MH "Schizophrenia Spectrum and Other Psychotic Disorders+") ) OR ( psychosis OR psychoses OR schizophrenia OR schizophrenia spectrum disorder* OR psychotic disorder* OR non-affective psychosis OR schizo* OR schizoaffective disorder* ) ) AND ( ( (MH "Psychotherapy+") OR (MM "Psychosocial Intervention") OR (MH "Cognitive Behavioral Therapy+") ) OR ( psychological treatment OR psychotherapy OR psychological intervention OR psychosocial intervention OR therapy OR therapies OR treatment OR abreaction OR acceptance and commitment therapy OR acting out OR adlerian OR analytical psychotherapy OR analytical psychotherapies OR anger control OR anger management OR animal therapy OR animal therapies OR art therapy OR art therapies OR assertive training OR assertiveness training OR attention training technique OR autogenic training OR autosuggestion OR aversion therapy OR aversion therapies OR balint group OR befriending OR behavior contracting OR behavior modification OR behavior regulation OR behavior therapy OR behavior therapies OR behaviour contracting OR behaviour modification OR behaviour regulation OR behaviour therapy OR behaviour therapies OR bibliotherapy OR bibliotherapies OR biofeedback OR body psychotherapy OR body psychotherapies OR brief psychotherapy OR brief psychotherapies OR caregiver support OR cbt OR client centre OR client center OR cognitive behavior OR cognitive behaviorial OR cognitive intervention OR cognitive interventions OR cognitive rehabilitation OR cognitive remediation OR cognitive technique OR cognitive techniques OR cognitive therapy OR cognitive therapies OR cognitive treatment OR cognitive treatments OR compassionate mind training OR conjoint therapy OR conjoint therapies OR contingency management OR conversational therapy OR conversational therapies OR conversion therapy OR conversion therapies OR coping skills OR counseling OR counselling OR countertransference OR couples therapy OR couples therapies OR covert sensitization OR covert sensitisation OR crisis intervention OR dance therapy OR dance therapies OR dialectic OR dialectical OR dream analysis OR eclectic OR emotion focused OR emotionally focused OR emotional freedom technique OR encounter group therapy OR encounter group therapies OR existential therapy OR existential therapies OR experiential psychotherapy OR experiential psychotherapies OR exposure therapy OR exposure therapies OR expressive psychotherapy OR expressive psychotherapies OR eye movement desensitization OR eye movement desensitisation OR family intervention OR family interventions OR family therapy OR family therapies OR feminist therapy OR feminist therapies OR free association OR freudian OR geriatric psychotherapy OR geriatric psychotherapies OR gestalt therapy OR gestalt therapies OR griefwork OR group intervention OR group interventions OR group psychotherapy OR group psychotherapies OR group therapy OR group therapies OR guided imagery OR holistic psychotherapy OR holistic psychotherapies OR humanistic psychotherapy OR humanistic psychotherapies OR hypnosis OR hypnotherapy OR hypnotherapies OR hypnotizability OR hypnotisability OR imagery OR implosive therapy OR implosive therapies OR individual psychotherapy OR individual psychotherapies OR insight therapy OR insight therapies OR integrated psychological therapy OR integrative psychotherapy OR integrative psychotherapies OR integrative therapy OR integrative therapies OR interpersonal OR jungian OR kleinian OR logotherapy OR marathon group therapy OR marathon group therapies OR marital therapy OR marital therapies OR meditation OR mental healing OR metacognitive therapy OR metacognitive therapies OR metacognitive training OR milieu therapy OR milieu therapies OR mindfulness OR morita therapy OR morita therapies OR multimodal OR music therapy OR music therapies OR narrative therapy OR narrative therapies OR nondirective therapy OR nondirective therapies OR object relations OR person centred therapy OR person centred therapies OR person centered therapy OR person centered therapies OR personal construct therapy OR personal construct therapies OR persuasion therapy OR persuasion therapies OR pet therapy OR pet therapies OR play therapy OR play therapies OR primal therapy OR primal therapies OR problem solving OR psychoanalyse OR psychoanalysed OR psychoanalysis OR psychoanalytic OR psychodrama OR psychodynamic OR psychoeducate OR psychoeducation OR psychoeducating OR psychologic OR psychological OR psychologically OR psychological therapy OR psychological therapies OR psychosocial treatment OR psychosocial treatments OR psychotherapy OR psychotherapies OR psychotherapeutic counsel OR psychotherapeutic counseling OR psychotherapeutic counselling OR psychotherapeutic processes OR psychotherapeutic training OR psychotherapeutic treatment OR psychotherapeutic treatments OR rational emotive OR reality therapy OR reality therapies OR reciprocal inhibition OR rehabilitation OR rehabilitating OR relationship therapy OR relationship therapies OR relaxation OR reminiscence therapy OR reminiscence therapies OR rogerian OR role play OR role plays OR role playing OR self analysis OR self analysing OR self esteem OR sensitivity training OR sex therapy OR sex therapies OR sleep phase chronotherapy OR sleep phase chronotherapies OR social skills education OR social skills training OR socioenvironmental therapy OR socioenvironmental therapies OR sociotherapy OR solution focused OR stress management OR support group OR support groups OR supportive therapy OR supportive therapies OR systematic desensitization OR systematic desensitisation OR systemic therapy OR systemic therapies OR therapeutic community OR therapeutic communities OR transactional analysis OR transference OR transtheoretical OR validation therapy OR validation therapies ) ) |  |
| #11 | #10 with Limiters - Publication Type: Adaptive Clinical Trial, Clinical Study, Clinical Trial, Controlled Clinical Trial, Randomized Controlled Trial |  |
| **PubMed** | | |
| *Search Number* | *Search Details* | *Results* |
| #10 | #3 AND #6 AND #9 |  |
| #9 | #7 OR #8 |  |
| #8 | psychological treatment[Title/Abstract] OR psychotherapy[Title/Abstract] OR psychological intervention[Title/Abstract] OR psychosocial intervention[Title/Abstract] OR therapy[Title/Abstract] OR therapies[Title/Abstract] OR treatment[Title/Abstract] OR abreaction[Title/Abstract] OR acceptance[Title/Abstract] AND commitment therapy[Title/Abstract] OR acting out[Title/Abstract] OR adlerian[Title/Abstract] OR analytical psychotherapy[Title/Abstract] OR analytical psychotherapies[Title/Abstract] OR anger control[Title/Abstract] OR anger management[Title/Abstract] OR animal therapy[Title/Abstract] OR animal therapies[Title/Abstract] OR art therapy[Title/Abstract] OR art therapies[Title/Abstract] OR assertive training[Title/Abstract] OR assertiveness training[Title/Abstract] OR attention training technique[Title/Abstract] OR autogenic training[Title/Abstract] OR autosuggestion[Title/Abstract] OR aversion therapy[Title/Abstract] OR aversion therapies[Title/Abstract] OR balint group[Title/Abstract] OR befriending[Title/Abstract] OR behavior contracting[Title/Abstract] OR behavior modification[Title/Abstract] OR behavior regulation[Title/Abstract] OR behavior therapy[Title/Abstract] OR behavior therapies[Title/Abstract] OR behaviour contracting[Title/Abstract] OR behaviour modification[Title/Abstract] OR behaviour regulation[Title/Abstract] OR behaviour therapy[Title/Abstract] OR behaviour therapies[Title/Abstract] OR bibliotherapy[Title/Abstract] OR bibliotherapies[Title/Abstract] OR biofeedback[Title/Abstract] OR body psychotherapy[Title/Abstract] OR body psychotherapies[Title/Abstract] OR brief psychotherapy[Title/Abstract] OR brief psychotherapies[Title/Abstract] OR caregiver support[Title/Abstract] OR cbt[Title/Abstract] OR client centre[Title/Abstract] OR client center[Title/Abstract] OR cognitive behavior[Title/Abstract] OR cognitive behaviorial[Title/Abstract] OR cognitive intervention[Title/Abstract] OR cognitive interventions[Title/Abstract] OR cognitive rehabilitation[Title/Abstract] OR cognitive remediation[Title/Abstract] OR cognitive technique[Title/Abstract] OR cognitive techniques[Title/Abstract] OR cognitive therapy[Title/Abstract] OR cognitive therapies[Title/Abstract] OR cognitive treatment[Title/Abstract] OR cognitive treatments[Title/Abstract] OR compassionate mind training[Title/Abstract] OR conjoint therapy[Title/Abstract] OR conjoint therapies[Title/Abstract] OR contingency management[Title/Abstract] OR conversational therapy[Title/Abstract] OR conversational therapies[Title/Abstract] OR conversion therapy[Title/Abstract] OR conversion therapies[Title/Abstract] OR coping skills[Title/Abstract] OR counseling[Title/Abstract] OR counselling[Title/Abstract] OR countertransference[Title/Abstract] OR couples therapy[Title/Abstract] OR couples therapies[Title/Abstract] OR covert sensitization[Title/Abstract] OR covert sensitisation[Title/Abstract] OR crisis intervention[Title/Abstract] OR dance therapy[Title/Abstract] OR dance therapies[Title/Abstract] OR dialectic[Title/Abstract] OR dialectical[Title/Abstract] OR dream analysis[Title/Abstract] OR eclectic[Title/Abstract] OR emotion focused[Title/Abstract] OR emotionally focused[Title/Abstract] OR emotional freedom technique[Title/Abstract] OR encounter group therapy[Title/Abstract] OR encounter group therapies[Title/Abstract] OR existential therapy[Title/Abstract] OR existential therapies[Title/Abstract] OR experiential psychotherapy[Title/Abstract] OR experiential psychotherapies[Title/Abstract] OR exposure therapy[Title/Abstract] OR exposure therapies[Title/Abstract] OR expressive psychotherapy[Title/Abstract] OR expressive psychotherapies[Title/Abstract] OR eye movement desensitization[Title/Abstract] OR eye movement desensitisation[Title/Abstract] OR family intervention[Title/Abstract] OR family interventions[Title/Abstract] OR family therapy[Title/Abstract] OR family therapies[Title/Abstract] OR feminist therapy[Title/Abstract] OR feminist therapies[Title/Abstract] OR free association[Title/Abstract] OR freudian[Title/Abstract] OR geriatric psychotherapy[Title/Abstract] OR geriatric psychotherapies[Title/Abstract] OR gestalt therapy[Title/Abstract] OR gestalt therapies[Title/Abstract] OR griefwork[Title/Abstract] OR group intervention[Title/Abstract] OR group interventions[Title/Abstract] OR group psychotherapy[Title/Abstract] OR group psychotherapies[Title/Abstract] OR group therapy[Title/Abstract] OR group therapies[Title/Abstract] OR guided imagery[Title/Abstract] OR holistic psychotherapy[Title/Abstract] OR holistic psychotherapies[Title/Abstract] OR humanistic psychotherapy[Title/Abstract] OR humanistic psychotherapies[Title/Abstract] OR hypnosis[Title/Abstract] OR hypnotherapy[Title/Abstract] OR hypnotherapies[Title/Abstract] OR hypnotizability[Title/Abstract] OR hypnotisability[Title/Abstract] OR imagery[Title/Abstract] OR implosive therapy[Title/Abstract] OR implosive therapies[Title/Abstract] OR individual psychotherapy[Title/Abstract] OR individual psychotherapies[Title/Abstract] OR insight therapy[Title/Abstract] OR insight therapies[Title/Abstract] OR integrated psychological therapy[Title/Abstract] OR integrative psychotherapy[Title/Abstract] OR integrative psychotherapies[Title/Abstract] OR integrative therapy[Title/Abstract] OR integrative therapies[Title/Abstract] OR interpersonal[Title/Abstract] OR jungian[Title/Abstract] OR kleinian[Title/Abstract] OR logotherapy[Title/Abstract] OR marathon group therapy[Title/Abstract] OR marathon group therapies[Title/Abstract] OR marital therapy[Title/Abstract] OR marital therapies[Title/Abstract] OR meditation[Title/Abstract] OR mental healing[Title/Abstract] OR metacognitive therapy[Title/Abstract] OR metacognitive therapies[Title/Abstract] OR metacognitive training[Title/Abstract] OR milieu therapy[Title/Abstract] OR milieu therapies[Title/Abstract] OR mindfulness[Title/Abstract] OR morita therapy[Title/Abstract] OR morita therapies[Title/Abstract] OR multimodal[Title/Abstract] OR music therapy[Title/Abstract] OR music therapies[Title/Abstract] OR narrative therapy[Title/Abstract] OR narrative therapies[Title/Abstract] OR nondirective therapy[Title/Abstract] OR nondirective therapies[Title/Abstract] OR object relations[Title/Abstract] OR person centred therapy[Title/Abstract] OR person centred therapies[Title/Abstract] OR person centered therapy[Title/Abstract] OR person centered therapies[Title/Abstract] OR personal construct therapy[Title/Abstract] OR personal construct therapies[Title/Abstract] OR persuasion therapy[Title/Abstract] OR persuasion therapies[Title/Abstract] OR pet therapy[Title/Abstract] OR pet therapies[Title/Abstract] OR play therapy[Title/Abstract] OR play therapies[Title/Abstract] OR primal therapy[Title/Abstract] OR primal therapies[Title/Abstract] OR problem solving[Title/Abstract] OR psychoanalyse[Title/Abstract] OR psychoanalysed[Title/Abstract] OR psychoanalysis[Title/Abstract] OR psychoanalytic[Title/Abstract] OR psychodrama[Title/Abstract] OR psychodynamic[Title/Abstract] OR psychoeducate[Title/Abstract] OR psychoeducation[Title/Abstract] OR psychoeducating[Title/Abstract] OR psychologic[Title/Abstract] OR psychological[Title/Abstract] OR psychologically[Title/Abstract] OR psychological therapy[Title/Abstract] OR psychological therapies[Title/Abstract] OR psychosocial treatment[Title/Abstract] OR psychosocial treatments[Title/Abstract] OR psychotherapy[Title/Abstract] OR psychotherapies[Title/Abstract] OR psychotherapeutic counsel[Title/Abstract] OR psychotherapeutic counseling[Title/Abstract] OR psychotherapeutic counselling[Title/Abstract] OR psychotherapeutic processes[Title/Abstract] OR psychotherapeutic training[Title/Abstract] OR psychotherapeutic treatment[Title/Abstract] OR psychotherapeutic treatments[Title/Abstract] OR rational emotive[Title/Abstract] OR reality therapy[Title/Abstract] OR reality therapies[Title/Abstract] OR reciprocal inhibition[Title/Abstract] OR rehabilitation[Title/Abstract] OR rehabilitating[Title/Abstract] OR relationship therapy[Title/Abstract] OR relationship therapies[Title/Abstract] OR relaxation[Title/Abstract] OR reminiscence therapy[Title/Abstract] OR reminiscence therapies[Title/Abstract] OR rogerian[Title/Abstract] OR role play[Title/Abstract] OR role plays[Title/Abstract] OR role playing[Title/Abstract] OR self analysis[Title/Abstract] OR self analysing[Title/Abstract] OR self esteem[Title/Abstract] OR sensitivity training[Title/Abstract] OR sex therapy[Title/Abstract] OR sex therapies[Title/Abstract] OR sleep phase chronotherapy[Title/Abstract] OR sleep phase chronotherapies[Title/Abstract] OR social skills education[Title/Abstract] OR social skills training[Title/Abstract] OR socioenvironmental therapy[Title/Abstract] OR socioenvironmental therapies[Title/Abstract] OR sociotherapy[Title/Abstract] OR solution focused[Title/Abstract] OR stress management[Title/Abstract] OR support group[Title/Abstract] OR support groups[Title/Abstract] OR supportive therapy[Title/Abstract] OR supportive therapies[Title/Abstract] OR systematic desensitization[Title/Abstract] OR systematic desensitisation[Title/Abstract] OR systemic therapy[Title/Abstract] OR systemic therapies[Title/Abstract] OR therapeutic community[Title/Abstract] OR therapeutic communities[Title/Abstract] OR transactional analysis[Title/Abstract] OR transference[Title/Abstract] OR transtheoretical[Title/Abstract] OR validation therapy[Title/Abstract] OR validation therapies[Title/Abstract] |  |
| #7 | "psychotherapy"[MeSH Terms] OR "psychosocial intervention"[MeSH Terms] OR "behavior therapy"[MeSH Terms] OR "behavior therapy"[MeSH Terms] OR "complementary therapies"[MeSH Terms] OR "psychoanalysis"[MeSH Terms] OR "counseling"[MeSH Terms] OR "psychoanalytic therapy"[MeSH Terms] OR "psychoanalytic therapy"[MeSH Terms] |  |
| #6 | #4 OR #5 |  |
| #5 | "psychosis"[Title/Abstract] OR "psychoses"[Title/Abstract] OR "schizophrenia"[Title/Abstract] OR "schizophrenia spectrum disorder*"[Title/Abstract] OR "psychotic disorder*"[Title/Abstract] OR "non affective psychosis"[Title/Abstract] OR "schizo*"[Title/Abstract] OR "schizoaffective disorder*"[Title/Abstract] |  |
| #4 | (schizophrenia[MeSH Terms]) OR (paranoid disorders[MeSH Terms]) |  |
| #3 | #1 OR #2 |  |
| #2 | "auditory hallucination*"[Title/Abstract] OR "hallucination*"[Title/Abstract] OR "verbal auditory hallucination*"[Title/Abstract] OR "hearing voice*"[Title/Abstract] |  |
| #1 | hallucinations[MeSH Terms] |  |
| **Cochrane Library** | | |
| *Search Number* | *Search Details* | *Results* |
| #1 | MeSH descriptor: [Schizophrenia] explode all trees |  |
| #2 | MeSH descriptor: [Schizophrenia Spectrum and Other Psychotic Disorders] explode all trees |  |
| #3 | MeSH descriptor: [Psychotic Disorders] explode all trees |  |
| #4 | (schizo* OR psychotic disorder*):ti,ab,kw |  |
| #5 | #1 OR #2 OR #3 OR #4 |  |
| #6 | MeSH descriptor: [Hallucinations] explode all trees |  |
| #7 | (auditory hallucination* OR hallucination* OR verbal auditory hallucination* OR hearing voice*) |  |
| #8 | #6 OR #7 |  |
| #9 | MeSH descriptor: [Psychotherapy] explode all trees |  |
| #10 | MeSH descriptor: [Psychosocial Intervention] explode all trees |  |
| #11 | MeSH descriptor: [Behavior Therapy] explode all trees |  |
| #12 | MeSH descriptor: [Cognitive Behavioral Therapy] explode all trees |  |
| #13 | MeSH descriptor: [Psychoanalysis] explode all trees |  |
| #14 | MeSH descriptor: [Counseling] explode all trees |  |
| #15 | (psychological treatment OR psychotherapy OR psychological intervention OR psychosocial intervention OR therapy OR therapies OR treatment OR abreaction OR acceptance and commitment therapy OR acting out OR adlerian OR analytical psychotherapy OR analytical psychotherapies OR anger control OR anger management OR animal therapy OR animal therapies OR art therapy OR art therapies OR assertive training OR assertiveness training OR attention training technique OR autogenic training OR autosuggestion OR aversion therapy OR aversion therapies OR balint group OR befriending OR behavior contracting OR behavior modification OR behavior regulation OR behavior therapy OR behavior therapies OR behaviour contracting OR behaviour modification OR behaviour regulation OR behaviour therapy OR behaviour therapies OR bibliotherapy OR bibliotherapies OR biofeedback OR body psychotherapy OR body psychotherapies OR brief psychotherapy OR brief psychotherapies OR caregiver support OR cbt OR client centre OR client center OR cognitive behavior OR cognitive behaviorial OR cognitive intervention OR cognitive interventions OR cognitive rehabilitation OR cognitive remediation OR cognitive technique OR cognitive techniques OR cognitive therapy OR cognitive therapies OR cognitive treatment OR cognitive treatments OR compassionate mind training OR conjoint therapy OR conjoint therapies OR contingency management OR conversational therapy OR conversational therapies OR conversion therapy OR conversion therapies OR coping skills OR counseling OR counselling OR countertransference OR couples therapy OR couples therapies OR covert sensitization OR covert sensitisation OR crisis intervention OR dance therapy OR dance therapies OR dialectic OR dialectical OR dream analysis OR eclectic OR emotion focused OR emotionally focused OR emotional freedom technique OR encounter group therapy OR encounter group therapies OR existential therapy OR existential therapies OR experiential psychotherapy OR experiential psychotherapies OR exposure therapy OR exposure therapies OR expressive psychotherapy OR expressive psychotherapies OR eye movement desensitization OR eye movement desensitisation OR family intervention OR family interventions OR family therapy OR family therapies OR feminist therapy OR feminist therapies OR free association OR freudian OR geriatric psychotherapy OR geriatric psychotherapies OR gestalt therapy OR gestalt therapies OR griefwork OR group intervention OR group interventions OR group psychotherapy OR group psychotherapies OR group therapy OR group therapies OR guided imagery OR holistic psychotherapy OR holistic psychotherapies OR humanistic psychotherapy OR humanistic psychotherapies OR hypnosis OR hypnotherapy OR hypnotherapies OR hypnotizability OR hypnotisability OR imagery OR implosive therapy OR implosive therapies OR individual psychotherapy OR individual psychotherapies OR insight therapy OR insight therapies OR integrated psychological therapy OR integrative psychotherapy OR integrative psychotherapies OR integrative therapy OR integrative therapies OR interpersonal OR jungian OR kleinian OR logotherapy OR marathon group therapy OR marathon group therapies OR marital therapy OR marital therapies OR meditation OR mental healing OR metacognitive therapy OR metacognitive therapies OR metacognitive training OR milieu therapy OR milieu therapies OR mindfulness OR morita therapy OR morita therapies OR multimodal OR music therapy OR music therapies OR narrative therapy OR narrative therapies OR nondirective therapy OR nondirective therapies OR object relations OR person centred therapy OR person centred therapies OR person centered therapy OR person centered therapies OR personal construct therapy OR personal construct therapies OR persuasion therapy OR persuasion therapies OR pet therapy OR pet therapies OR play therapy OR play therapies OR primal therapy OR primal therapies OR problem solving OR psychoanalyse OR psychoanalysed OR psychoanalysis OR psychoanalytic OR psychodrama OR psychodynamic OR psychoeducate OR psychoeducation OR psychoeducating OR psychologic OR psychological OR psychologically OR psychological therapy OR psychological therapies OR psychosocial treatment OR psychosocial treatments OR psychotherapy OR psychotherapies OR psychotherapeutic counsel OR psychotherapeutic counseling OR psychotherapeutic counselling OR psychotherapeutic processes OR psychotherapeutic training OR psychotherapeutic treatment OR psychotherapeutic treatments OR rational emotive OR reality therapy OR reality therapies OR reciprocal inhibition OR rehabilitation OR rehabilitating OR relationship therapy OR relationship therapies OR relaxation OR reminiscence therapy OR reminiscence therapies OR rogerian OR role play OR role plays OR role playing OR self analysis OR self analysing OR self esteem OR sensitivity training OR sex therapy OR sex therapies OR sleep phase chronotherapy OR sleep phase chronotherapies OR social skills education OR social skills training OR socioenvironmental therapy OR socioenvironmental therapies OR sociotherapy OR solution focused OR stress management OR support group OR support groups OR supportive therapy OR supportive therapies OR systematic desensitization OR systematic desensitisation OR systemic therapy OR systemic therapies OR therapeutic community OR therapeutic communities OR transactional analysis OR transference OR transtheoretical OR validation therapy OR validation therapies):ti,ab,kw |  |
| #16 | #9 OR #10 OR #11 OR #12 OR #13 OR #14 OR #15 |  |
| #17 | #5 AND #8 AND #16, in Trials |  |

*Note*. The presented examples only serve as an overview about the data base search that will be modified during the data process.
